# Qualitative Study on the Factors Influencing the Utilisation of Products Labelled “Food for Special Medicinal Use” (FSMP)

**DOI:** 10.3390/nu15112582

**Published:** 2023-05-31

**Authors:** Marius Calin Chereches, Hajnal Finta, Cristian Olimpiu Popa, Daniela Stefanescu, Daniela-Lucia Muntean

**Affiliations:** 1 Faculty of Pharmacy, “George Emil Palade” University of Medicine, Pharmacy, Science and Technology of Târgu Mureș, 540142 Târgu Mureș, Romania; hajnal.finta@umfst.ro (H.F.); daniela.muntean@umfst.ro (D.-L.M.); 2 Faculty of European Studies, Babeș-Bolyai University, 400090 Cluj-Napoca, Romania; cris.popa@gmail.com; 3 Faculty of Economics and Law, “George Emil Palade” University of Medicine, Pharmacy, Science and Technology of Târgu Mureș, 540142 Târgu Mureș, Romania; daniela.stefanescu@umfst.ro

**Keywords:** food for special medical purposes, FSMP, qualitative research, nutritional needs of oncology patients

## Abstract

This study aimed to investigate Romanian physicians’ awareness, recommendation practices, and opinions regarding the use of Foods for Special Medical Purposes (FSMPs) products. A total of ten physicians were interviewed using a structured questionnaire, and their responses were analysed using thematic content analysis. The study found that physicians were aware of FSMPs and recommended them to their patients based on nutritional deficits, weight loss, or deglutition impairments. In addition, disease stage, treatment scheme, taste, affordability, and availability were identified as factors influencing the recommendation and use of FSMPs. While physicians generally did not consult clinical trials, clinical experience was deemed essential for recommending FSMPs to patients. Patients’ feedback regarding the usage and sourcing of FSMPs was generally positive, with some expressing concerns about the availability of different flavours and the costs of purchasing the products. This study concluded that physicians play a vital role in recommending FSMPs to patients and ensuring they have the necessary nutritional support during treatment. However, it may be imperative to consider the provision of additional patient education materials and fostering collaborative efforts with nutritionists in order to optimise the prospects of positive outcomes in oncology treatment, while simultaneously alleviating the financial burdens faced by patients.

## 1. Introduction

Food for special medical purposes (FSMPs) are foods designed for people with particular medical disorders and nutritional needs. They are often only available with a prescription and are meant to be used under medical supervision. FSMPs can come in different forms and can be used in various settings, such as hospitals, long-term care facilities, and home settings. Examples of medical conditions that may call for the use of FSMPs include inborn errors of metabolism, severe food allergies, and gastrointestinal illnesses.

The role of physicians in recommening he use of FSMPs is pivotal. When dealing with these products, medical doctors encounter complexity concerning legislation, composition, special regulation, and compliance factors, including formulation and pricing. This research aims to qualitatively explore doctors’ recommendations for FSMP products through in-depth interviews.

### 1.1. Definition and Legislation Applicable to FSMPs

To ensure their effectiveness and safety, FSMPs are governed by regulations and are required to adhere to specific standards. The FSMP industry is regulated by various national and international bodies, such as the European Food Safety Authority (EFSA) and the U.S. Food and Drug Administration (FDA), which set standards for the safety, quality, and efficacy of FSMP products [1,2,3,4].

FSMPs are defined according to Article 2(2)(g) of Regulation 609/2013 [5] as food specially processed or formulated for the dietary management of patients, including infants, to be used under medical supervision. It is intended for exclusive or partial feeding of patients with a limited capacity to take, digest, absorb, metabolise, or excrete ordinary food or certain nutrients.

For example, in Romania, Regulation 128/2018 updated Regulation 609/2013 mentioned above (named within most of the documents as “FSG Regulation”), and the food notification procedure for special medical purposes (FSMP) was set by Order of the Ministry of Health nr.820/2019 [6]. Furthermore, specific standards that apply to FSMP are also outlined within Codex Alimentarius by the World Health Organisation. At the same time, the Commission clarified the distinction between FSMP and food supplement products within a dedicated document [1,4].

### 1.2. Understanding FSMP Composition and Labelling Requirements

FSMPs are divided into three groups [1]: (a) nutritionally complete foods with a standard nutrient formulation, (b) nutritionally complete foods with a nutrient-adapted formulation unique to a disease, disorder, or medical condition, and (c) nutritionally incomplete foods with either a standard formulation or a nutrient-adapted formulation unique to a disease, disorder, or medical condition. The first two categories can serve as a patient’s only source of nutrition, whereas the third can only increase intake to supplement other sources of nutrients. The FSG Regulation and Codex Alimentarius both clearly define the composition of products, which are defined as FSMP that must contain specific nutrients from the List (vitamins, minerals, amino acids, carnitine-taurine, nucleotides, and choline-inositol) or meet specific requirements (meet food standards, provide scientific support regarding the contribution of ingredients to satisfying nutritional needs and to comply with conditions related to pesticides) [2,4,5].

Some writers believe that FSMPs intended for oncology patients may not be adequate for some critical elements, such as Zn, Cu, Se, Fe, or Mn, despite the regulatory standards regarding the composition of FSMPs, implying that the formulations are based on commonly accepted scientific facts [7]. In addition, similar studies have indicated that sample determination for Se, Zn, K, Mg, and vitamin C differ from (E.U.) 2016/128 requirements for enteral tube feed formulae, raising questions about the bioavailability of synthetic vitamins and minerals in comparison to those from natural sources [8].

Products classified as FSMP should be usable by humans and provide nutritional support, while a different classification is being developed by an interdisciplinary group in the German industry [5,9].

Regulatory bodies have highlighted the importance of accurately labelling FSMP products. Labelling should provide information regarding energy value, protein content, vitamins and minerals, osmolarity and/or acid-base balance, and the origin and nature of proteins. Manufacturers should disclose the nature of the product, its purpose, and specific usage instructions in sufficient detail. References to usage guidelines should be made, such as use only under medical supervision and not suited for parenteral use. It is important to provide educational materials and resources to healthcare professionals [1,4,10].

### 1.3. Examining the Use of FSMPs in Cancer Care: Treatment Benefits and Associated Costs

Stomatitis and oral mucositis are common in oncology patients receiving chemotherapy or radiation therapy, which can have a negative impact on their quality of life. Nutrition can help reduce stress and oncology treatment expenses.

Medical costs for patients receiving radiotherapy and developing mucositis or pharyngitis during the treatment are much greater than for those without such conditions, according to a study on patients with head and neck cancer (HNC) and non-small cell lung cancer (USD39,313 vs. USD20,798) [11]. Another study on HNC patients receiving radiotherapy found that 91 percent of patients experienced oral mucositis, and 66 percent of those cases were severe (>Grade 3). Depending on the severity, oral mucositis was associated with increased expenses of USD1700–USD6000 [12]. In addition, the side effects of cancer treatment, such as nausea, vomiting, oral mucositis, and exhaustion, create numerous logistical difficulties and significantly strain the patient and the healthcare system, especially when cancer is treated as a chronic condition [13]. According to a different study, oral or/and G.I. mucositis occurred in 51% of chemotherapy-treated cancer patients with solid tumours or lymphoma. If a patient has oral mucositis or both, G.I. and oral mucositis, the projected cost of chemotherapy for each cycle is 1.6 times higher and 2.3 times higher, respectively. For patients receiving a haematopoietic cell transplant, an additional point in the peak mucositis score resulted in an additional USD25,000 in hospital expenses [14]. In treating several cancers, surgery, chemotherapy (including immunotherapy or target therapy), and radiotherapy may be employed, and the expenditures involved with all these efforts are substantial [15].

Early nutrition support and prevention of radiochemotherapy-related side effects for cancer patients can reduce weight loss, fewer breaks, delays, and hospitalisations, and increase treatment completion [14,16,17,18].

National healthcare systems reimburse FSMP products in several countries. The E.U. allows for regional or provincial variations in granting FSMP reimbursement. France, Germany, Italy, Spain, and the UK are some nations that reimburse FSMPs. Additionally, France and Brazil are the countries that have a formal HTA process for medical nutrition [19]. However, FSMP type and content may affect reimbursement criteria and procedures. It is critical to remember that depending on the country and the patient’s medical condition, different restrictions may apply for FSMP product reimbursement [19,20,21]. Chinese researchers and authorities place a high value on FSMP and health technology assessment (HTA—a systematic evaluation of healthcare interventions to determine their safety, effectiveness, and cost-effectiveness) for products under this category. Several papers have been written about creating rules, clinical trial prerequisites, and HTA evaluation standards [22,23,24].

### 1.4. Exploring the Landscape of FSMP Products: Market Size, Key Brands, Players, and Unique Marketing Factors

The worldwide FSMP market is estimated to be worth USD11.2 billion in 2019 or USD13.48 billion in 2021, and could reach USD19.67 billion in 2028 or USD19.41 billion in 2030. The key drivers of growth are increased awareness of the benefits of this category of products, increased prevalence of chronic diseases, increased demand for older adults, proliferation of new private label manufacturers, and expansion of new distribution channels. More than 65 percent of the market share is held by the top 10 companies, including Nestle, Danone (Nutricia), Abbott, Bayer, Mead-Johnson, Ajinomoto, Fresenius-Kabi, Lenus Pharma, GFI-Gruppo Farmaimpresa, Galen Limited, BOSSD, and Leskon & EnterNutr [25,26,27].

The regulation stipulates that using FSMPs requires medical supervision, and healthcare professionals (HCPs) can help patients with FSMPs. Without the limits typically imposed on communication to the general public, HCPs must receive complete information regarding the product composition, clinical justification, appropriate usage, preparation, and intended target group [5,20]. Pharmacies and pharmacists must investigate the legal responsibilities for dispensing FSMPs, as there are no formal prescription requirements to trace the recommendation process [28].

Public outreach efforts should be limited to labelling, educational content, and using digital techniques like Search Engine Optimisation (SEO) and Search Engine Marketing (SEM). Digitalisation of healthcare systems is a fact that will significantly impact future issues. Digital tools can help monitor, assess, and manage food safety hazards and offer consumers and health professionals information and transparency. Additionally, digital technologies can assist in customising FSMPs based on an individual’s needs [29].

## 2. Material and Methods

The research technique examined was in-depth interviews with medical professionals with specialities in the sectors that suggest patients with demands for such products—cancer, ENT, radiation, or surgery—to evaluate the factors influencing the recommendation and use of FSMPs. The in-depth interview method allowed the researcher to approach the subject with common questions. It enables us to decide which issues the participants should be questioned about during the quantitative research phase. Using this strategy, we can obtain a wealth of descriptive information on people’s attitudes, behaviours, and perceptions [30]. The following benefits of in-depth interview methods [15] are beneficial for this study:

- Since respondents are dispersed, bringing them together for a focus group study would be difficult and expensive.
- While a respondent has approximately 10 min in a focus group setting, and the other participants may influence his perspective, in an in-depth interview, only 30 to 45 min are set aside for the conversation with one respondent.
- The debate may disclose previously assumed habits since the participants fully engage with the topic and tell the whole tale.
- Since using FSMPs requires specialised expertise, the study process necessitates thorough explanations of some subjects.
- Later, the research agenda incorporates respondents’ viewpoints.

The operators were given instructions on conducting themselves before and during the interviews. The logic of the research was emphasised, as well as simple instructions such as the need for a preparatory discussion before the interview, how to conduct the interview itself and to follow the guide, as well as how notes should be made, and finally, the extraction of answers and the final document for each subject. 

The interview outline contained several subjects for conversation with the issues, including:

Is the subject recommending FSMPs? Do they know the category of FSMP? How did they become aware of this category?

Which factors influence the subject’s recommendation? Clinical proof, the stage of the disease, correlation with the underlying treatment, and relationships with other specialities?

Which particular names or brands are popular or used? What features should the items that the subject recommends or uses have?

Does the subject know what laws apply to FSMPs? Did the topic have any thoughts on how to make clarity more precise?

Price: Are FSMPs expensive or affordable? Who covers the cost of the products? 

Who are the suppliers, and what channels procure FSMP products? 

The opinions of physicians about patients’ feedback about the use of FSMPs.

Subject’s feedback, in their medical capacity.

Some data about the subject of the interview to analyse the answers, such as speciality, type of medical unit in which they work, city, and age, experience, or associations with academic work.

The operators were additionally equipped with the following:

Script of introductory talk with the subject about the research, a short description of the research aims, and a little information about FSMPs.

A list of the most popular brands/products available to help the subjects put the questions into context.

Three operators conducted in-depth interviews, and answers from 10 interviews were collected. The interviews were conducted from October 2022 to January 2023.

Our hypothesis for this qualitative research was that doctors would assess a multitude of factors prior to recommend Food for Special Medical Purposes (FSMPs), including:

- The patient’s medical condition: FSMPs are designed for those with particular medical conditions with unique dietary requirements. Doctors will assess the patient’s condition to decide whether an FSMP is necessary.
- Nutrient requirements: FSMPs are created to satisfy a patient’s unique nutrient requirements. Doctors will consider the patient’s nutritional demands and determine whether an FSMP can fulfil those needs.
- Digestive capabilities: FSMPs are designed for those whose medical circumstances prevent them from following a typical diet. Doctors will assess the patient’s digestibility and decide whether an FSMP is necessary.
- Allergies or intolerances: Patients with severe food allergies or intolerances may take FSMPs. If an FSMP is necessary, doctors will assess the patient’s allergies or food intolerances.
- Medical history: The doctor’s recommendation of an FSMP may also consider the patient’s medical background. For instance, to guarantee enough nutrition, a patient with a history of malabsorption may need an FSMP.
- Accessibility: Doctors will also consider the patient’s ability to access the FSMP. This encompasses elements such as price, accessibility, and usability.

## 3. Results and Discussion

Ten in-depth interviews were conducted to gather information, and the distribution of specialities and places of residency are shown in the accompanying Table 1:

Key findings were grouped following this article’s interview guidelines and narrative logic.

### 3.1. Awareness about the FSMP Category of Products

All subjects interviewed were aware of FSMP products.

When asked where they first learned about FSMPs, most respondents (7/10) reported that they got their information from the producers’ medical personnel, and only one said they found out on their own through an internet search. Two respondents stated that they knew these items from their training period for their internship or residency more than ten years prior. The other doctors—their colleagues, who learned from medical reps, were an additional source of information. The statement made by one respondent that information about FSMPs was provided at the medical committee’s weekly meetings, where medical representatives attend and present various goods, was also noteworthy. Other respondents addressed the distribution of samples to patients. The Fresibin, Nutridrink, and Nutricia lines were cited in response to brands.

When we enquired about FSMP information sources, the responses were consistent. They all listed the same kinds of resources: medical representatives, inserts, and pamphlets ordered by the manufacturers, documents found online through searches, and various manufacturing company presentations at medical congresses and events.

### 3.2. How Are These Products Recommended?

The conditions surrounding the recommendations of FSMPs were crucial to our study. Respondents mentioned that patients with specific illnesses (pancreatic cancer, gastric cancer, oesophageal cancer, bronchopulmonary cancer, and any cancer of ENT kind) are candidates of choice to benefit from FSMP use. Due to the prolonged length of combo treatment, nutritional support with FSMPs is especially necessary for cancer of the ENT type (chemotherapy and radiotherapy). According to one respondent’s experience, patients who utilised FSMPs during their treatment likely improved the treatment and had greater success. Several respondents mentioned nutritional deficits, weight loss, or deglutition impairments (dysphagia or xerostomia). According to that respondent, one responder further linked the use of FSMPs with the clinic’s nutritionist, who suggests that such products be used. All respondents mentioned that they recommended FSMP products to their patients. Patients were encouraged to use FSMPs after being discharged from the hospital for those who required such indication.

### 3.3. Which Factors Influence the Recommendations?

Usually, the doctor makes the recommendation, and the availability of products in the pharmacies in the neighbourhood of the hospital or patient’s residence is considered.

Another respondent was more specific, saying that in ENT cancers, the standard is to recommend FSMPs starting with the 2nd week of treatment, which takes six weeks, and the patient is monitored every week concerning weight and alimentation.

In addition, it was reported that cancer patients and those with Chronic obstructive pulmonary disease (COPD) had difficulty with deglutition or nutrition and appeared cachectic. Therefore, these products were also advised for their use. 

Most respondents mentioned that patients with cancers in ENT, oesophagus, bronchopulmonary, or patients with widespread metastasis and palliative needs require FSMPs to keep their nutritional status, weight, and general condition satisfactory.

### 3.4. Are Clinical Trials Available to Influence the Recommendations?

Most respondents mentioned that they did not consult clinical trials, some justifying due to lack of time. However, some respondents emphasised that clinical experience is essential for recommending FSMP products to patients. At the same time, one respondent considered that there was no significant difference between various brands within this category of products. It is worth mentioning that one respondent considered that clinical trials are available and results are good ones and recommends using such products in line with the stage of the disease. However, the same doctor recommends keeping the patient without medicinal products and with ordinary alimentation as long as possible.

### 3.5. Does the Disease Stage Influence the Choice of FSMPs?

Respondents generally indicated weight loss and body mass index (BMI) as principal triggers for recommending FSMPs to patients under treatment. The primary need is to have the patient well balanced from a nutritional point of view to support chemo or radiotherapy treatment for the duration. FSMP products help patients to achieve this objective. The appearance of problems with deglutition or food absorption also triggered the recommendation of using these products, as well as certain medical conditions (such as gastrectomy, oesophagectomy, acute pancreatitis, or complete dysphagia for solid food).

A subject stressed that, when making a suggestion, affordability must be addressed alongside medical reasons.

### 3.6. Is the Recommendation Correlated with the Treatment Scheme?

FSMPs are recommended for cancers, such as colon cancer or pancreatic cancer, with the condition that makes ordinary alimentation challenging to achieve, according to one respondent or the recommendation being linked with BMI or weight loss.

Another respondent considered that the administration has nothing to do with the treatment scheme but with the comfort or the patient’s needs, so the FSMPs might be recommendable to those in need.

The link with the treatment is that doctors must ensure that the patient can follow the treatment scheme. Particularly in radiotherapy, postponing or delaying treatment is not an option. Thus, FSMP products may help the patient cope with the side effects of chemo or radiotherapy and enable him to keep up with and complete the whole treatment scheme successfully. 

### 3.7. Which Products or Brands Are Used or Recommended?

The respondents spontaneously mentioned four brands, as per the Table 2:

In addition to brand names, several respondents cited other requirements for items to be utilised, including hypercaloric or normocaloric content, high protein content, and various tastes and flavours.

### 3.8. What Features and Characteristics of Products Make Them Useful?

Taste and flavour were the most significant and commonly cited characteristics. According to the feedback received by the physicians in our panel, some patients claimed that certain products were overly sweet or had a high level of acidity. In contrast, others disapproved of products that tasted like bananas. Conversely, patients more readily accepted FSMPs when they tasted like strawberries and chocolate. 

One respondent gave a more detailed description, emphasising the demand for products that are lactose-free, gluten-free, high in vitamins, minerals, and proteins, and hypercaloric.

### 3.9. Is the Pertinent Legal Framework Known? Are There Any Necessary Changes That Need to Be Made?

With one exception, our respondents were unaware of whether there was specific legislation governing FSMPs in place and how this product category differed from food supplements. One participant had an in-depth knowledge of E.U. regulations, informational requirements, informational requirements for the public and specialists, classification, quality standards, labelling, indication, and precautions. No change to the current legislation was mentioned as necessary, identified, or proposed by participants in this research. 

### 3.10. Price and Affordability

Participants paid careful attention to concerns regarding the pricing and accessibility of FSMPs, which were identified as an important topic affecting the use and availability of FSMPs for in-need patients.

Three respondents indicated their awareness of prices; one accurately identified the average bottle price as RON 15, but the other two knew a price range of RON 30–50. One even brought up the substantial distinction between Nutridrink, an example of a more reasonably priced product with RON 30 /bottle, and Fresubin, an example of an expensive product with RON 50/bottle. According to respondents’ calculations, a patient would require products costing between RON 30 and 50 for every bottle (RON 4–5000 or EUR 800–1000), which is out of reach for many patients, given the overall cost of cancer therapy.

We gathered some pricing for products in this category from online pharmacies or wholesalers and their spot prices to explain the mathematics of the price problem. The The collected information can be found in Table 3.

Using the price data from the above sources, the cost for a patient using two bottles daily for seven weeks would range from RON 1433 to 2948 (i.e., EUR 290–590). Furthermore, the National Institute of Statistics, cited by Statista, said that the average salary in Romania was RON 3416 in 2021, which is necessary to make the previous information pertinent. 

The responses from the other respondents were less detailed. At the same time, 1/3 of them said that these products are generally accessible and affordable, and the other third claimed that they are far too expensive for patients to afford to be used as they should be. 

### 3.11. Who Is Paying for FSMP Products?

In Romania, FSMPs are not reimbursed; consequently, the patient, their family, or the hospital covers the expense of these items during hospitalisation. At the same time, most treatments are also administered in an outpatient setting, and most respondents to our survey said that the patient is responsible for purchasing these goods. There were two mentions of the hospital purchasing such products for hospitalised patients, out of the general budget of the healthcare unit, or it received donations. Respondents also emphasised that for some patients, the cost of purchasing the required quantity of FSMP products may exceed their financial means.

### 3.12. How Do Patients Source FSMP Products?

As far as our respondents learned from personal experience, buying these products from pharmacies, online pharmacies, or online shops doesn’t present any significant problems. However, some stressed the necessity of placing a preorder to guarantee that the required versions would be available. One participant spoke about successfully obtaining Medidrink from the online pharmacy Spring Farma and buying FSMP products online rather than from traditional pharmacies or the neighbouring country, Hungary, which may be more affordable in some cases. Pharmacy, online shopping, and charity donations were the supply methods highlighted. One participant brought up a hospital-organised tender for the purchase of Fresubin.

Additionally, we examined the market data from the research firm Cegedim [31], which compiles data from hospitals and retail pharmacies in Romania, as shown in the following Table 4.

For 2022, we identified reports of relatively low sales and only a few brands. Although this is the only source we could find, it is improbable that only 106,178 units were sold through retail pharmacies in Romania. We can only speculate that online pharmacies are not counted and that most online shops did not report sales to Cegedim, thus underestimating the totals. Only Fresubin indicates large sales among the important manufacturers who are present on the market with more brands than respondents to our research reported (over 90 percent of the total).

### 3.13. What Feedback Did the Responders Receive from Their Patients?

We can group the feedback on two main aspects—how patients use the products and how they can source them.

Patients are generally happy with the products. However, patients are concerned about the availability of different flavours to suit specific needs in terms of usage, especially in the case of some patients whose tastes are altered during chemotherapy or radiotherapy. In addition, it is important to note that certain patients expressed concerns about items being either too sweet or acidic in some circumstances.

Regarding sourcing, some patients expressed the necessity of placing an order in advance, asking family members for information, or looking up sources online. However, the biggest problem mentioned is that most patients need help to secure the funding necessary to buy the supplements during their therapy, especially in cases of a 6–7 week treatment scheme associated with the condition requiring FSMP aid.

### 3.14. The Opinions of the Physicians Regarding FSMPs

The most important objective for the participants in this research concerning the use of FSMP products is to help patients go through the course of treatment in an acceptable physical shape, and nutritional status is paramount for such an aim. For example, one participant in the study reported that FSMP users were less likely to have stomatitis or mucositis. In addition, most survey respondents said that patients were better equipped nutritionally to withstand the effects of chemotherapy and/or radiotherapy over their entire course.

Since some patients could not drink coffee during therapy, suggestions such as adding a coffee-like flavour were made. Since the financial burden currently exists for cancer patients, the added expense of FSMP products creates enormous obstacles for patients. Hence, respondents to our poll emphasised the necessity for a method to pay these costs externally through reimbursement or a similar mechanism.

Doctors emphasise that these expenses (i.e., for FSMP) are less than what would be needed for chemotherapy or radiation therapy. Since this keeps the patients well enough to complete the course successfully, the treatments are unquestionably worthwhile.

### 3.15. Extra Details Provided by Respondents

We asked respondents about any specific subjects they would like to bring up after the interview, and the following we believe merits discussion:

Using the FSMP would lower the cost of patient care while being necessary for finishing the treatment. 

It might be beneficial to commission more patient education materials on FSMP.

The help and collaboration of a nutritionist are critical since they free up the doctor from this responsibility and assist patients at risk.

In some extreme cases (i.e., N.G. tube feeding), using FSMPs would lower the burden on the budget necessary for treatment and the patient’s comfort.

### 3.16. Limitations

While the responses from our participants demonstrated overall convergence on key topics, it is crucial to acknowledge the limitations associated with the small sample size and the specific focus on physicians specialising in oncology-related medical fields. Additionally, it is important to note that participant bias is a common limitation inherent in qualitative research. Therefore, to enhance the depth and breadth of understanding regarding the utilisation of FSMP products, further research incorporating larger sample sizes, a broader range of medical specialities, and alternative methodologies such as focus group setups would significantly contribute valuable insights to the field.

## 4. Conclusions

This study’s findings provide insight into Romanian physicians’ awareness, recommendation practices, and opinions regarding the use of FSMPs. Physicians recommended FSMPs to patients based on specific medical conditions, treatment schemes, taste preferences, and affordability. Disease stage and treatment scheme were identified as factors that influenced the recommendation and use of FSMPs. Clinical experience was deemed essential for recommending FSMPs to patients, while patients’ feedback regarding the usage and sourcing of FSMPs was generally positive. This study concluded that physicians play a crucial role in ensuring that patients have the necessary nutritional support during treatment. However, enhancing patient education materials and fostering collaboration with nutritionists could be necessary to enhance the likelihood of positive outcomes in oncology treatment and alleviate the financial burden on patients. Overall, the study highlights the importance of FSMPs in patient care and the need for further research and collaboration among healthcare professionals to optimise the use of these products.

## Figures and Tables

**Table 1:** Demographics of the respondents.

Residence		Medical Speciality	
Brasov	2	ENT’s	1
Cluj Napoca	2	Oncology	4
Constanța	1	Pneumology	1
București	1	Radiotherapy	3
Oradea	1	Surgeron	1
Sibiu	1		
Târgu Mures	2		

**Table 2:** FSMP brands mentioned by respondents (count of mentions).

MediDrink	1/10
Nutridrink	3/10
Fresubin	9/10
Nutricia	1/10

**Table 3:** Price information about brands mentioned by respondents and sources of information.

Product	List Price per Pack	Source of Information	Bottles per Pack	Price per Bottle	Price per 7 Weeks Treatment (2 Bottles/Day)
Fresubin Protein Energy, 4 × 200 mL	RON 62.88	https://alimentespeciale.ro/fresubin-protein-energy-drink-fructe-tropicale-x-200mL-fresenuis-kabi/103593.htm (accesed on 2 March 2023)	4	RON 15.72	RON 1540.53
Bautura cu aroma de vanilie Fresubin 2 kcal, 4 × 200 Ml—hypercaloric	RON 80.02	https://alimentespeciale.ro/fresubin-2kcal-drink-x-200ml-vanilie-fresenius-kabi/100992.htm (accesed on 2 March 2023)	4	RON 20.01	RON 1960.49
Fresubin Pro Drink Alune de padure 4 × 200 mL—hypercaloric, hyper-proteic	RON 120.33	https://alimentespeciale.ro/fresubin-pro-drink-alune-de-padure-4x200ml-fresenius-kabi/161630.htm (accesed on 2 March 2023)	4	RON 30.08	RON 2948.09
Fresubin, 4 × 200 mL, Fresenius Kabi Germania	RON 57.50	https://comenzi.farmaciatei.ro/vitamine-si-suplimente/digestie/nutritie-speciala/bautura-energizanta-cu-proteine-aroma-de-vanilie-fresubin-4x200-ml-fresenius-kabi-germania-p323667 (accesed on 2 March 2023)	4	RON 14.38	RON 1408.75
MediDrink plus vanilie × 200 mL	RON 17.00	https://www.farmaciilenapofarm.ro/nutritie-speciala/medidrink-plus-vanilie-x200ml-39202.html (accesed on 2 March 2023)	1	RON 17.00	RON 1666.00
Nutridrink banane × 200 mL Nutricia	RON 14.62	https://alimentespeciale.ro/nutridrink-banane-x-200ml-nutricia/103021.htm (accesed on 2 March 2023)	1	RON 14.62	RON 1432.76

Sources for prices have been accessed on 2 March 2023.

**Table 4:** Sales (units and value) for FSMP on pharmacy channels in Romania for the year 2022 (source: Cegedim) PPP = pharmacy purchase price.

Manufacturer	Product Name	Sales 2022 (Units)	Value 2022 (PPP, RON)
**FRESENIUS-KABI**		**106,178**	**4,807,822**
	FRESUBIN	92,786	4,032,172
	DIBEN	4692	203,146
	SUPPORTAN	4220	279,809
	SURVIMED	2722	165,364
	PROVIDEXTRA DRINK	663	44,565
	CALSHAKE	502	19,499
	FREBINI	453	18,469
	KABI GLUTAMINE	140	44,798
**NESTLE**		**21,532**	**790,416**
	OPTIFIBRE	20,971	749,973
	NUTREN OPTIMUM	420	20,571
	MODULEN	141	19,872
**NUTRICIA**		**2408**	**25,558**
	NUTRIDRINK	2408	25,558

## Data Availability

The data presented in this study are available on request from the corresponding author.
